# Distinctive facial features in idiopathic Moyamoya disease in Caucasians: a first systematic analysis

**DOI:** 10.7717/peerj.4740

**Published:** 2018-06-27

**Authors:** Markus Kraemer, Quoc Bao Huynh, Dagmar Wieczorek, Brunilda Balliu, Barbara Mikat, Stefan Boehringer

**Affiliations:** 1 Department of Neurology, Alfried Krupp Hospital Essen, Essen, Germany; 2 Department of Neurology, University Clinic of Duesseldorf, Duesseldorf, Germany; 3 Institute of Human Genetics, University of Duesseldorf, Duesseldorf, Germany; 4 Institute of Human Genetics, University of Essen, Essen, Germany; 5 Institute of Genetics, Stanford University School of Medicine, Stanford, CA, United States of America; 6 Biomedical Data Sciences, Leiden University Medical Center, Leiden, The Nederlands

**Keywords:** Moyamoya disease, Idiopathic, Resemblance, Face, Genetic causes, Photographs

## Abstract

**Background:**

Craniofacial dysmorphic features are morphological changes of the face and skull which are associated with syndromic conditions. Moyamoya angiopathy is a rare cerebral vasculopathy that can be divided into Moyamoya syndrome, which is associated or secondary to other diseases, and into idiopathic Moyamoya disease. Facial dysmorphism has been described in rare genetic syndromes with associated Moyamoya syndrome. However, a direct relationship between idiopathic Moyamoya disease with dysmorphic facial changes is not known yet.

**Methods:**

Landmarks were manually placed on frontal photographs of the face of 45 patients with bilateral Moyamoya disease and 50 matched controls. After procrustes alignment of landmarks a multivariate, penalized logistic regression (elastic-net) was performed on geometric features derived from landmark data to classify patients against controls. Classifiers were visualized in importance plots that colorcode importance of geometric locations for the classification decision.

**Results:**

The classification accuracy for discriminating the total patient group from controls was 82.3% (*P*-value = 6.3×10^−11^, binomial test, a-priori chance 50.2%) for an elastic-net classifier. Importance plots show that differences around the eyes and forehead were responsible for the discrimination. Subgroup analysis corrected for body mass index confirmed a similar result.

**Discussion:**

Results suggest that there is a resemblance in faces of Caucasian patients with idiopathic Moyamoya disease and that there is a difference to matched controls. Replication of findings is necessary as it is difficult to control all residual confounding in study designs such as ours. If our results would be replicated in a larger cohort, this would be helpful for pathophysiological interpretation and early detection of the disease.

## Introduction

Craniofacial dysmorphic features are morphological changes of the face and skull which are associated with syndromic conditions. Distinct characteristic values of individual faces can be assigned to certain constellations of dysmorphic features typical or even pathognomonic for some diseases. For clinical diagnosis the constellation of dysmorphic features is crucial and often allows visual diagnosis. Dysmorphic features of the face and skull are typical in Cornelia de Lange syndrome, Williams-Beuren syndrome and fragile X syndrome (Winter, 1996). In scientific literature some genetic syndromes are described in which a Moyamoya phenomenon is accompanied with typical morphological abnormalities of the face (Bakri et al., 1999; Taskintuna et al., 2003; Heyer et al., 2006; Girirajan et al., 2007; Miskinyte et al., 2011; Kilic et al., 2012).

Moyamoya angiopathy is a rare vasculopathy characterized by bilateral progressive narrowing and occlusion of the intracranial portion of the internal carotid artery, the middle cerebral artery and the anterior cerebral artery (Guey et al., 2015). Epidemiological data do not exist in Europe. Main manifestations in childhood or early adulthood are strokes or cerebral bleedings. Often earlier transient ischemic attacks, headaches or choreatiform movements are not recognized as severe warning symptoms before disabling manifestation (Kraemer et al., 2012a; Kraemer et al., 2016; Kraemer et al., 2017). This “orphan disease” is more often recognized in Asians, especially in Japan and Korea. However, it also appears in Caucasians. In Europe and the USA it is very rare, epidemiology data are not known. Per the definition, Moyamoya disease (MMD) is the term for bilateral disease without any other manifestation former thought being idiopathic (Fukui, 1997; Bersano et al., 2016). In contrast to this, Moyamoya syndrome (MMS) is associated with other diseases or states like neurofibromatosis type 1, trisomy 21, vasculitis or states after skull radiation (Fukui, 1997; Guey et al., 2015).

The above mentioned association of facial dysmorphisms are only described in MMS, especially in genetic syndromes (Guey et al., 2015) like trisomy 21, loss of BRCC3 deubiquitinating enzyme (Miskinyte et al., 2011), Smith-Magenis syndrome (Girirajan et al., 2007), PHACE(S) syndrome (Heyer et al., 2006), Majewski osteodysplastic primordial dwarfism type II (MOPD II) (Kilic et al., 2012), Seckel syndrome (Rahme et al., 2010) and Alagille syndrome (Rocha et al., 2012). Facial dysmorphism associated with MMS seems to be caused by the syndromes MMS is associated with (such as Smith-Magenis syndrome Girirajan et al., 2007) and not part of the MMS spectrum.

A direct relationship between idiopathic Moyamoya disease with dysmorphic facial changes is not known yet. The only study which assumes to an association between Moyamoya disease and cephalocephalic parameters is from Qureshi et al. (2014). In Quereshi’s study 13 patients who are named as having Moyamoya disease are compared with 39 matched controls (Qureshi et al., 2014). This working group found a significantly greater bi-parietal diameter, a significantly greater fronto-occipital diameter and a shorter distance between bregma and occiput in Moyamoya disease patients compared with controls. However, it has to be criticized that those patients with angiographically confirmed Moyamoya angiopathy were defined as idiopathic Moyamoya disease just in absence of trisomy 21. No other clinical data like CSF data or exclusion of NF1 or other genetic syndromes were found in this article.

Thus, this study was intended to clarify if also in those patients with Moyamoya disease thought to be idiopathic due to detailed exclusion of differential diagnoses distinctive facial features could be found. Detecting any typical distinctive facial features also in MMD and not only in rare genetic syndromes with MMS would allow potential pathophysiologic conclusions and would enable physician to recognize this rare disease earlier.

## Materials and Methods

The study was approved by the local ethics committee (no.: 03-2357) and informed written consent was obtained from all patients. A total of 45 individuals with MMD diagnosed as idiopathic participated in this study. All patients were examined via MRI and conventional angiography. Adult patients with bilateral idiopathic MMD listed in the database of the Moyamoya outpatient clinic at the Alfried-Krupp Hospital in Essen, Germany were considered for the present study. The patients met the diagnostic criteria of the “Research Committee on Spontaneous occlusion of the Circle of Willis” of the Japanese Ministry of Health (Fukui, 1997). Patients with Moyamoya syndrome were excluded. Moyamoya syndrome was diagnosed in patients with the following conditions or diseases: after cerebral radiotherapy, cerebral vasculitis, atherosclerosis, Down syndrome and Neurofibromatosis 1. Moreover, exclusion criteria included known concomitant diseases defining Moyamoya syndrome associated with facial abnormality such as MOPD II. All patients earlier underwent detailed examinations including transesophageal sonography, CSF, serum studies and skin examination to exclude other known reasons for MMS.

The patients were contacted per phone or email. For the acquisition of the photos instructions were sent to patients on how to take photos by themselves and sent in photos were checked for quality. We list the instructions in the appendix. In further cases photos were taken in the hospital on regular consultation (Alfried Krupp Hospital, Essen, Germany).

The patient group was matched with the control group on basic demographic variables such as age, gender and ethnicity. Controls were recruited from an earlier study and gave informed consent for the use of their facial data in studies concerning syndromic facial traits. The instructions for photography was the same like in patients as listed in the appendix.

The Essen cohort was described earlier (Boehringer et al., 2011b).

In addition, controls were also recruited through announcements in public places such as public offices, local labor, and citizen offices. In total 50 caucasian controls were recruited.

The mean age was 42.16 (median 41, SD 13.92) years. Nine male and 36 female patients were enrolled. All patients and controls were comparable in age and gender (see Table 1). All had Caucasian family background and came from central Europe mainly Germany. Clinical features of the patients are described in Table 2.

**Table 1:** Demographic characteristics of study participants.

	Patients (*n* = 45)	Controlls (*n* = 50)
Males	9	11
Females	36	39
Age (mean)	42.16	42.06
Age (median)	41	41
Age (standard deviation)	13.92	14.33

**Table 2:** Clinical features.

Clinical feature	Percent of patients affected
Symptom: transient ischemic attack	60%
Symptom: stroke	73%
Symptom: headaches	31%
Symptom: cerebral bleeding	6%
Livedo racemosa	11%
Arterial hypertension	36%

**Figure 1:**
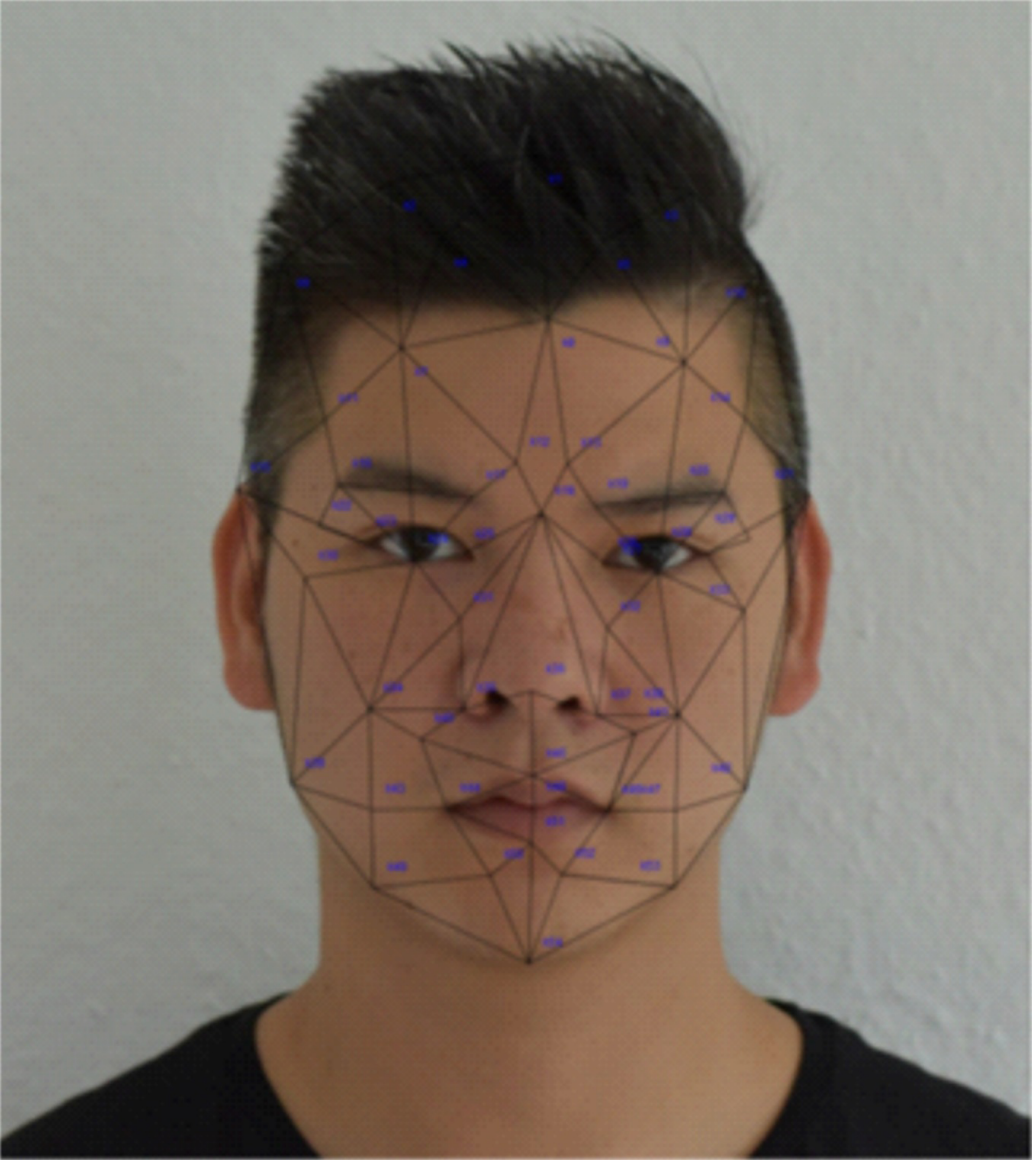
This example graph shows the author (QBH) with the Landmarks used in the study. In contrast to the author, all patients and controls were of Caucasian family background.

Landmarking of all facial photographs was performed manually by a single rater (QBH). A graph containing 31 landmarks was used and is shown in Fig. 1. Both patients and controls were of Caucasian family background.

As discussed below, confounding may play an important role in this study. To control for possible confounding due to high BMI, which might influence apparent resemblance of the face and might be more prevalent in the MMD group, a subgroup analysis was done. To this end, a subset of patients with BMI lower than 25 was compared to controls.

### Statistical analysis

We used methods described previously to analyze the data (Boehringer et al., 2011a; Balliu et al., 2014; Günther et al., 2015). In short, landmarks were manually placed on frontal photographs of patients and controls. A procrustes analysis was performed to align landmarks across photographs and a multivariate, penalized logistic regression was performed to classify patients against controls. Regressions were computed using elastic net regression as implemented in the R package *glmnet*. Penalization was necessary due to the high number of predictor variables (Trevor Hastie & Friedman, 2009). We used elastic net regression that allows to estimate models when there are more predictors than observations and simultaneously selects predictors, *i.e.* omits variables not necessary for prediction (Trevor Hastie & Friedman, 2009). Predictor variables were computed as follows: Input features for this regression were based on transformations of the landmark coordinates. First, 2D-coordinates were used as resulting from the Procrustes analysis. Secondly, pairwise distances between all landmarks were computed. Third, a Delaunay triangulation of the landmarks was computed and areas of the resulting triangles were computed. Fourth, angles within all these triangles were computed. Finally, all these values were combined in a vector being used as predicting variables in the penalized regression. The graph used contained 31 nodes and resulted in 62 coordinates, 741 pairwise distances, 47 areas, and 141 angles, resulting in a total of 991 features per photograph. An example (the author QBH) is shown in Fig. 1.

Classification accuracy was calculated using cross-validation and classifiers were visualized using importance plots. Importance plots are a color-coded representation of classifiers where for each point in the image the color represents how important features close to this point are for the classification decision. The value used for the color is computed by weighting absolute regression coefficient of a feature with the inverse of the distance of the feature to the point and summing up these values across all features to produce a single value per point. Finally, average images of the group were superimposed with these color representations (Balliu et al., 2014). Details of the procedures can be found in the cited literature (Boehringer et al., 2011a).

To increase reliability, the same rater (QBH) has done landmarking of the face photos for a second time to compare the results. We compared landmarks coordinates of the control group between the two batches with the same penalized regression. The classifier could distinguish the two batches almost perfectly (accuracy 98%) indicating high intra-rater variability. Classification was based on 27 features, which is less than the number of features found in classifiers for the Moyamoya phenotype (76 and 161). The *P*-value for the classification accuracy is a binomial test comparing observed with a-priori accuracy. It was calculated using R version 3.1.3 using function *binom.test* and a one-sided alternative.

## Results

**Figure 2:**
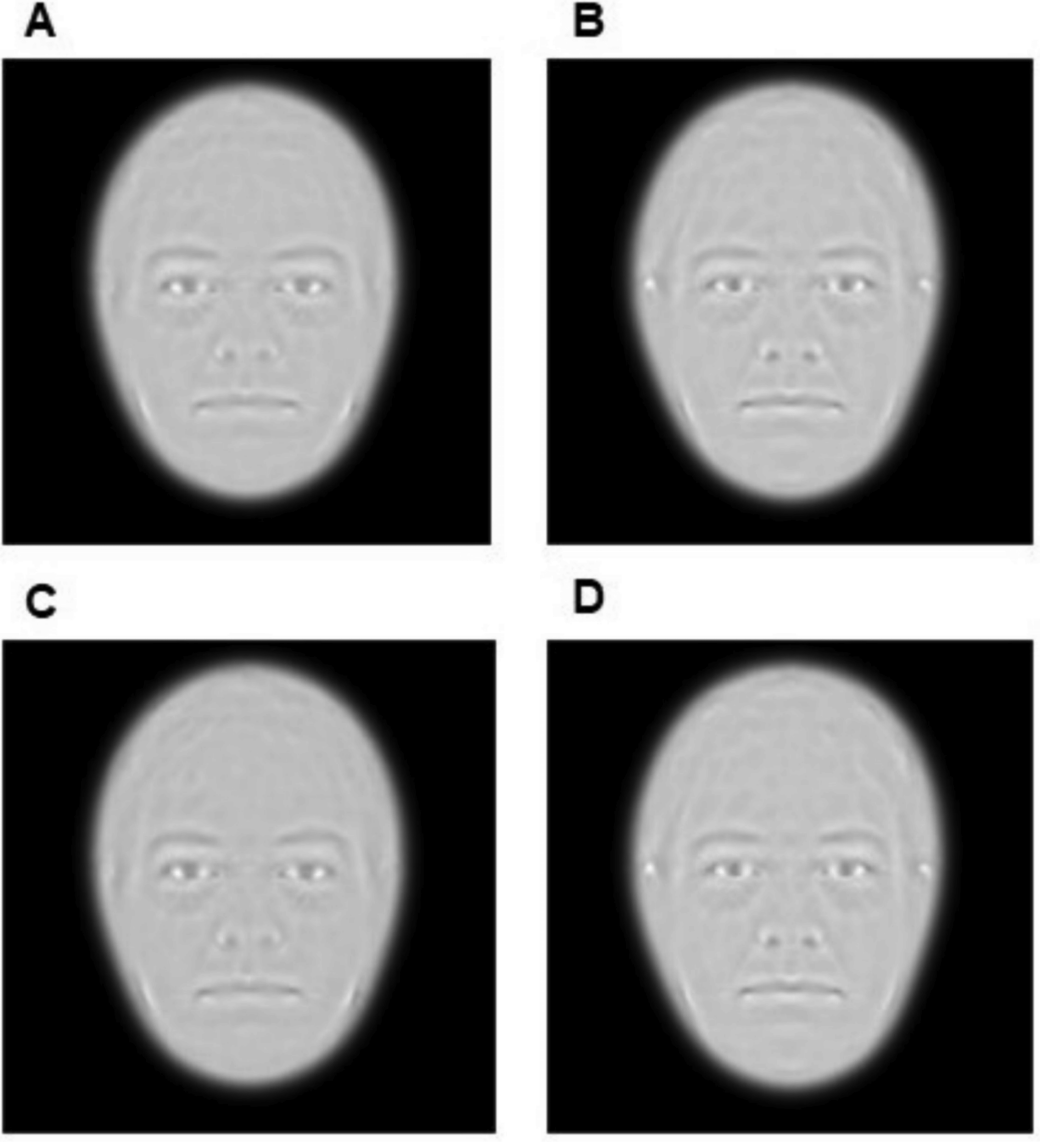
Average images of all Moyamoya patients (A) and all controls (B). Average images of patients (C) and controls (D) with low BMI.

Averages of the patient and control groups are shown in Fig. 2. Averages for the total patient group and low BMI patient group seem very subtle. In addition, patient and control group look very similar.

**Figure 3:**
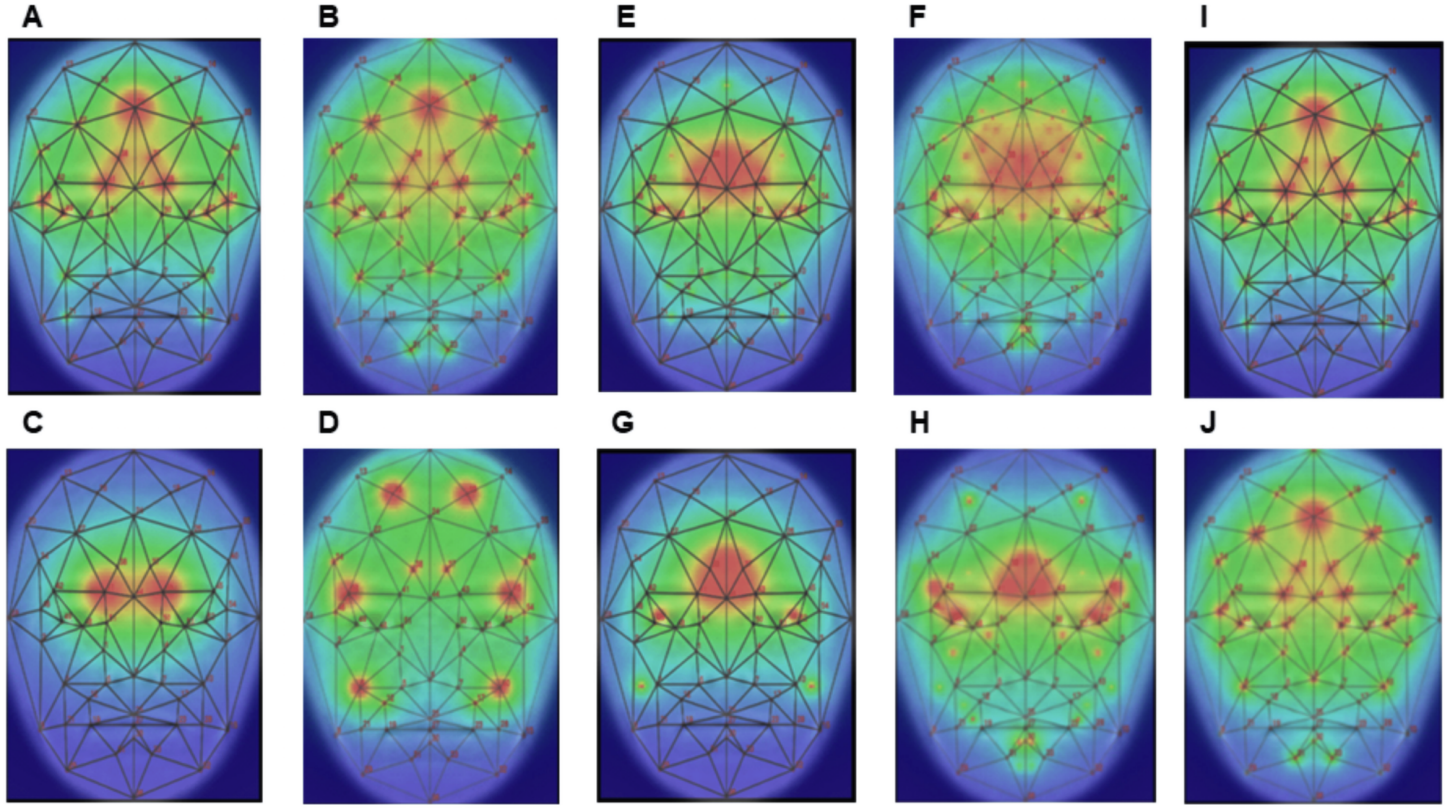
Importance plots, visualizing the classifiers. (A) All features, all patients; (B) all features, low BMI; (C) coordinates, all patients; (D) coordinates, low BMI; (E) distances, all patients; (F) distances, low BMI; (G) areas, all patients; (H) areas, low BMI; (I) angles, all patients; (J) angles, low BMI.

The classification accuracy for discriminating the total patient group from controls was 82.3% (*P*-value = 6.3 ×10^−11^, binomial test, a-priori chance 50.2%) for a mixing-parameter of 0.05 for the elastic-net classifier. A total of 76 features were selected to achieve the discrimination (Table 3). The importance plots show that differences around the eyes and forehead were responsible for the discrimination. Changes in angles seem to dominate the discrimination as the pattern exhibited by angles dominates the importance plot combining all features (Fig. 3).

The classification accuracy for discriminating the low BMI patients from controls was 83.9% (*P*-value = 2.4 ×10^−10^, binomial test, a-priori chance 51.5%). Again, the mixing-parameter for the elastic-net classifier was 0.05 for the best model with 162 selected features (Table 3). The importance plots are very similar to the ones for discriminating the total patient group from controls.

**Table 3:** Number of selected features per category.

	Coordinate	Distance	Area	Angle
All	1	49	4	22
Low BMI	11	84	15	51

## Discussion

The pathophysiology of MMD remains unclear and is still a matter of controversy (Bersano et al., 2016). Nevertheless, the final common pathway seems to involve proliferation of smooth muscle cells and their migration from media to intima. This process is regulated by various growth factors (Bersano et al., 2016). Elevated levels of basic fibroblast growth factors, soluble adhesion molecules, cellular retinoic acid-binding protein I, and hepatocyte growth factor were found (Bersano et al., 2016). Apoptosis, as reported to be associated with elevated caspase-3, occurred in the media of the MCA of MMD patients (Takagi et al., 2006).

Pathophysiologically, the pathways in the cell cycle are of particular interest, notably the DNA maintenance and the DNA damage response/repair (DDR) (Achrol et al., 2009; Sigdel et al., 2013). The above mentioned Xq28 deletion in three families with MMS result in loss of BRCC3, which is described to entail a defective angiogenesis in zebrafish (Miskinyte et al., 2011). Pericentin mutations in Seckel disease and MOPD II, both systemic diseases associated with MMS, also argue for a crucial role of the DNA damage repair system in the pathophysiology of MMA (Sigdel et al., 2013). Radiation as a trigger for MMS does also underline this as a possible pathophysiological mechanism (Sigdel et al., 2013). It is reasonable to assume that these pathophysiological mechanisms might also play a role in the observed changes in faces.

The role of the RNF213 mutation in MMD is still unclear. In Asia, in 95% of familiar and 79% of sporadic MMD the RNF213 mutation p.R4810K could be detected as a founder mutation. In contrast to this, in Caucasians no similar major founder gene seems to exist. Nevertheless, there are hints for genetic contributing factors also in Caucasian patients (Shoemaker et al., 2015). The Caucasian cohort in total may be more heterogeneous than the Asian cohort. However, it remains unclear how this type of genotype could affect the mechanism of craniofacial dysmorphism.

Given the rare occurrence in Caucasians, it has been controversially discussed, if analyses of these collectives should include all patients with MMA (e.g., MMD, MMS and unilateral type of MMA) or should be restricted to patients with idiopathic bilateral MMD only (Kraemer et al., 2012b) and some studies show inaccurate terminology (Kraemer et al., 2012b). In general, incidence and prevalence data in Japan are restricted to idiopathic bilateral MMD exclusive of MMS, while those in Europe and in the US are often reported inclusing MMA and unilateral MMA (Kraemer, Heienbrok & Berlit, 2008). The strength of this study was to include only those patients in whom idiopathic etiology was established due to the established criteria (Fukui, 1997). Moreover, our cohort size of 45 patients with MMD compared with the cohort sizes in the former Asian studies seems to be adequate considering that MMD is a rare disease in Europe (Kraemer, Heienbrok & Berlit, 2008).

Results suggest that there is a resemblance in faces of Caucasian patients with idiopathic Moyamoya disease and that there is a difference to matched controls.

Facial appearance seems to be almost identical between the groups. Flipping forth and back between images reveals that they are not identical but differences are subtle. Statistically, importance plots help to pinpoint the differences as found by the classifier. Angles of the triangle (21-41-43), which contains the nasion, seem to most influence the classifier by noticing that the contribution of all features (Figs. 3A, 3B, all) most resembles the contribution of angles (Figs. 3I, 3J, angles).

However, results as outlined for classification results and the underlying importance plots need to be interpreted with caution. Although photograph acquisition was standardized by a protocol, it is impossible to control all possible confounding factors such as lighting, or subtle pose deviations (Vollmar et al., 2008; Boehringer et al., 2011a). An important caveat is that patient and controls were recruited at different time points. Manual labeling was performed in a short time window of one week to avoid systematic changes in landmark evaluation. Apart from the variables above, camera technology, photographer, and more could potentially lead to confounding. Ideally, patients and controls would be matched on all important confounders, such as age, sex, and BMI and photographs would be taken on each pair in a single session. This ideal situation could not be achieved. However, by standardizing photograph acquisition, second landmarking, investigating the influence of BMI, and relying on a single rater, we tried to control variability in the study. Our re-labeling experiment indicates that intra-rater labeling can exceed true effects. However, this re-labeling experiment was conducted more than a year after the initial labeling run, which easily allows for subtle re-interpretations of landmark positions. Also, the features that caused the differences between the coordinate sets was different from the set found for disease classification and relied on fewer features. Although we cannot exclude classification purely based on labeling errors, we believe that our results support a subtle but distinguishable phenotype. On the other hand, we expect that the classifiers developed here would perform worse on unseen individuals as compared to the study internal results (Boehringer et al., 2011a).

In conclusion, facial differences from a control population might be detectable in idiopathic MMD using statistical means. If this results could be replicated in a larger cohort this would be helpful for pathophysiological interpretation and early detection of the disease. However, it is hypothesized that the differences in facial characteristics compared with controls are clinically very subtle in Caucasian patients with MMD due to genetic heterogeneity. In contrast to Asian patients, in Caucasians MMD it is thought to be triggered by various genetic causes, which could or could not be associated with facial abnormalities.

## Conclusion

In conclusion, this study suggest that there is a resemblance in faces of Caucasian patients with idiopathic Moyamoya disease and that there is a difference to matched controls.

##  Supplemental Information

10.7717/peerj.4740/supp-1 Supplemental Information 1 Importance angle control

10.7717/peerj.4740/supp-2 Data S1 Raw data: importance control area

10.7717/peerj.4740/supp-3 Data S2 Raw data: importance control background

10.7717/peerj.4740/supp-4 Data S3 Raw data: importance control background

10.7717/peerj.4740/supp-5 Data S4 Raw data: importance control background area

10.7717/peerj.4740/supp-6 Data S5 Raw data: importance control background coordinate

10.7717/peerj.4740/supp-7 Data S6 Raw data: importance control coordinate

10.7717/peerj.4740/supp-8 Data S7 Raw data: importance control distance

10.7717/peerj.4740/supp-9 Data S8 Raw data: importance patient all

10.7717/peerj.4740/supp-10 Data S9 Raw data: patient angle

10.7717/peerj.4740/supp-11 Data S10 Raw data: importance patient distance

10.7717/peerj.4740/supp-12 Data S11 Raw data: importance patient coordinate

10.7717/peerj.4740/supp-13 Data S12 Raw data: importance background angle

10.7717/peerj.4740/supp-14 Data S13 Raw data: control face

10.7717/peerj.4740/supp-15 Data S14 Raw data: controls

10.7717/peerj.4740/supp-16 Data S15 Raw data demographics

## Additional Information and Declarations

### Data Availability

The following information was supplied regarding data availability: The raw data are provided in the Supplemental Files.
