# Supplementary figures and images for: Distinctive facial features in idiopathic Moyamoya disease in Caucasians: a first systematic analysis
Source: PeerJ. 2018 Jun 27;6:e4740. doi: 10.7717/peerj.4740 (PMC6029584; doi:10.7717/peerj.4740)

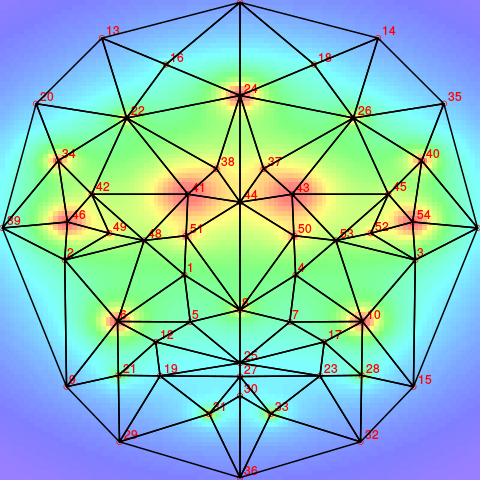

Supplement: Supplemental Information 1 [file peerj-06-4740-s001.png]

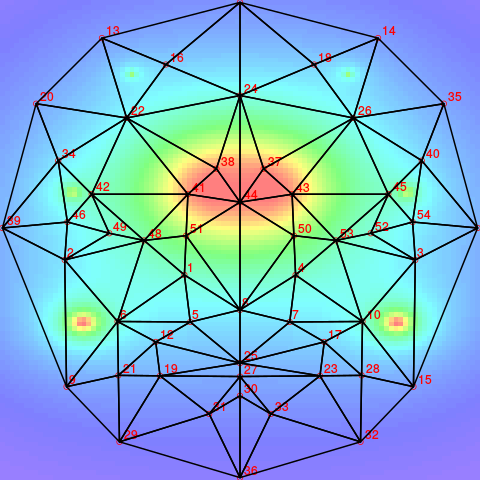

Supplement: Data S1 [file peerj-06-4740-s002.png]

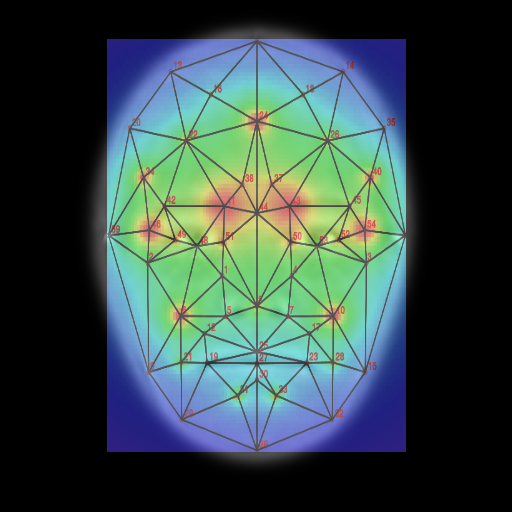

Supplement: Data S2 [file peerj-06-4740-s003.png]

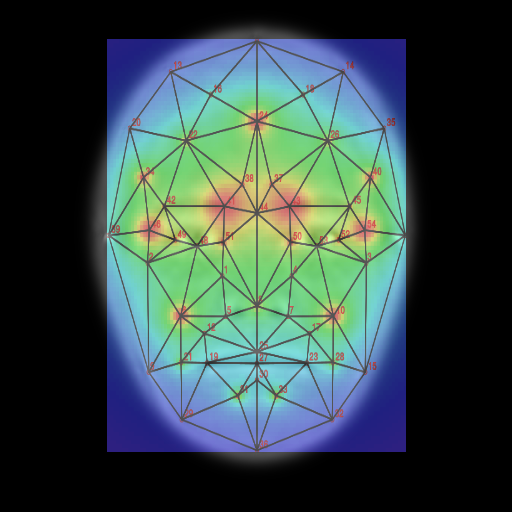

Supplement: Data S3 [file peerj-06-4740-s004.png]

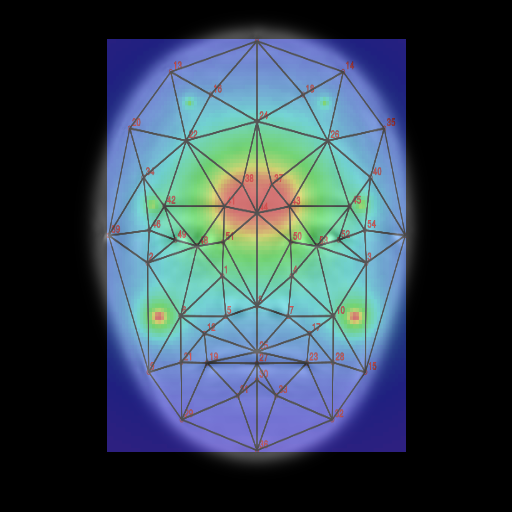

Supplement: Data S4 [file peerj-06-4740-s005.png]

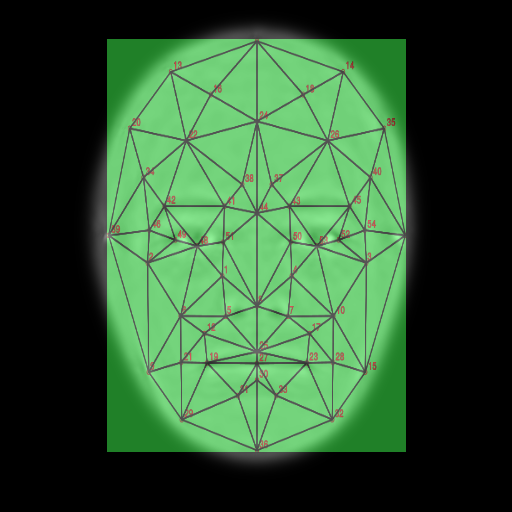

Supplement: Data S5 [file peerj-06-4740-s006.png]

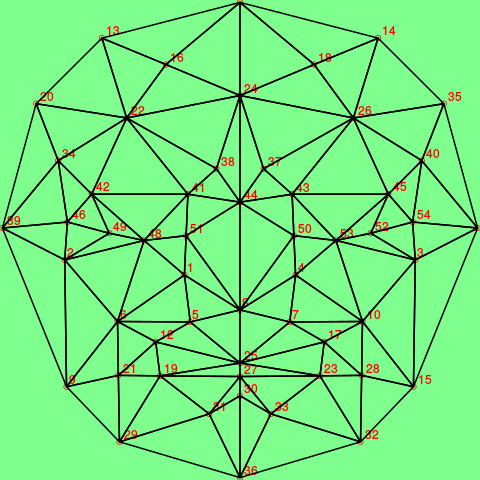

Supplement: Data S6 [file peerj-06-4740-s007.png]

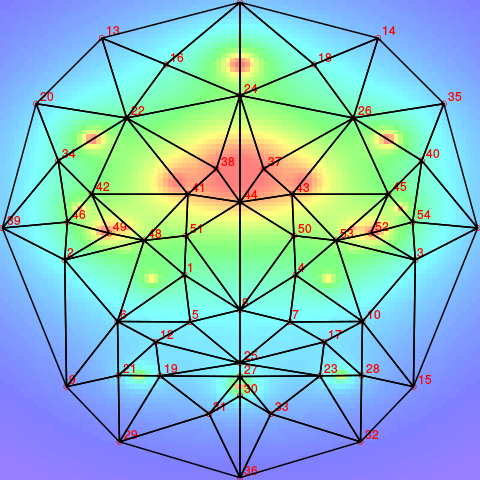

Supplement: Data S7 [file peerj-06-4740-s008.png]

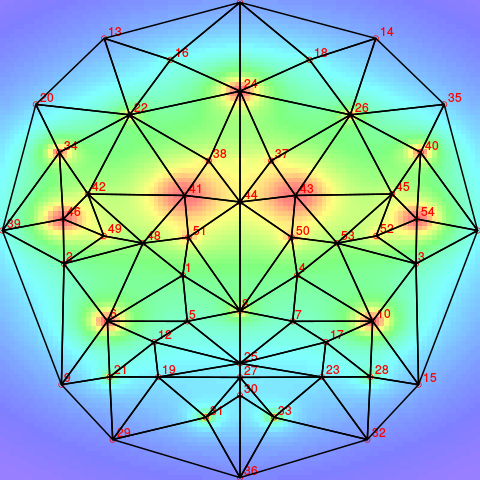

Supplement: Data S8 [file peerj-06-4740-s009.png]

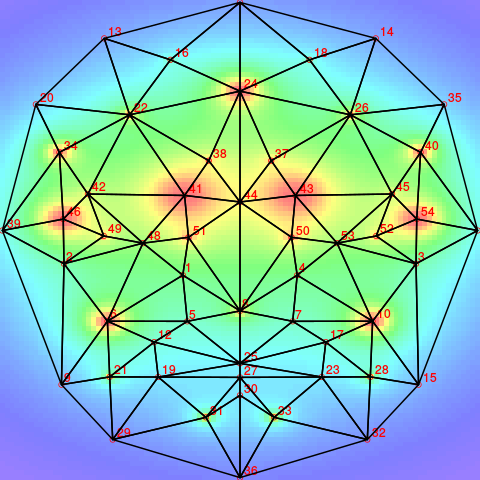

Supplement: Data S9 [file peerj-06-4740-s010.png]

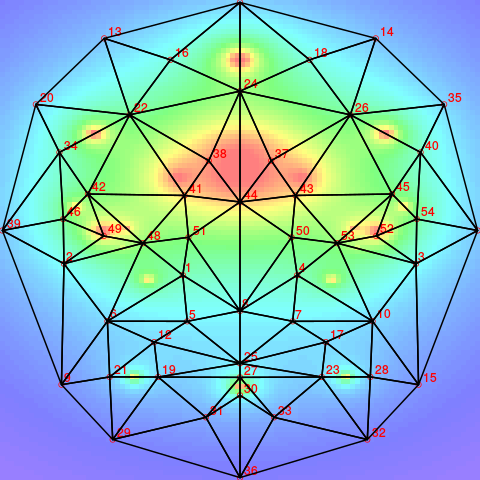

Supplement: Data S10 [file peerj-06-4740-s011.png]

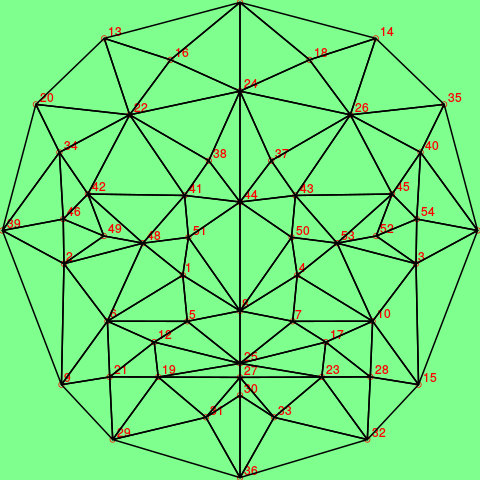

Supplement: Data S11 [file peerj-06-4740-s012.png]

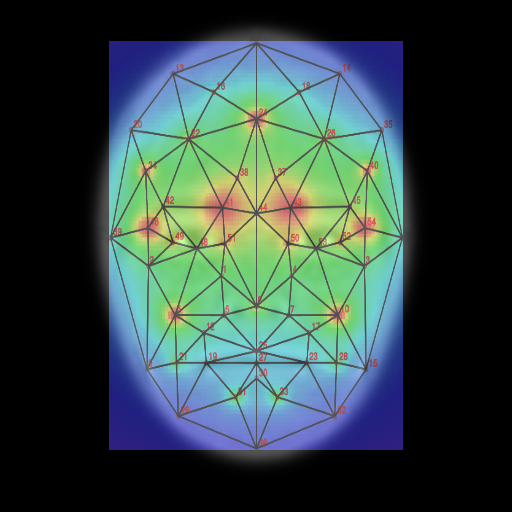

Supplement: Data S12 [file peerj-06-4740-s013.png]

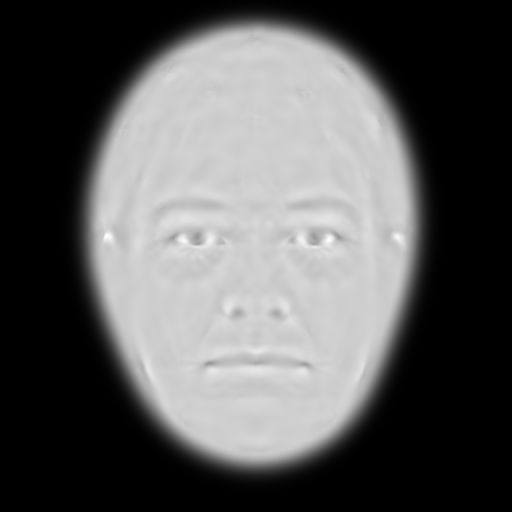

Supplement: Data S13 [file peerj-06-4740-s014.jpg]

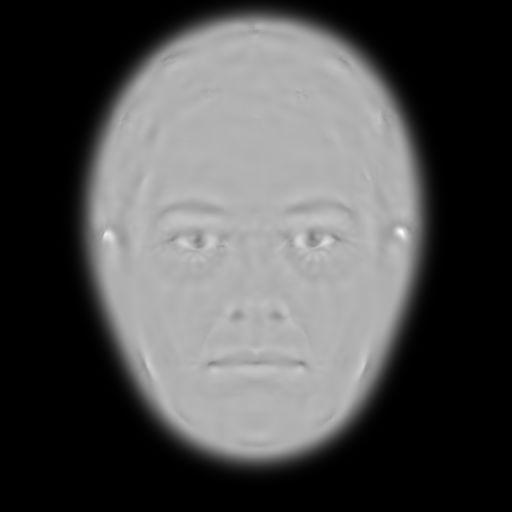

Supplement: Data S14 [file peerj-06-4740-s015.jpg]
